# Discovery and identification of potential anti-melanogenic active constituents of *Bletilla striata* by zebrafish model and molecular docking

**DOI:** 10.1186/s12906-021-03492-y

**Published:** 2022-01-07

**Authors:** Yiyuan Luo, Juan Wang, Shuo Li, Yue Wu, Zhirui Wang, Shaojun Chen, Hongjiang Chen

**Affiliations:** 1 College of Chinese Medicine, Zhejiang Pharmaceutical College, Ningbo, 315100 China; 2 College of Pharmacy, Nanjing University of Chinese Medicine, Nanjing, 210046 China

**Keywords:** *Bletilla striata*, Antioxidant, Anti-melanogenic activity, UPLC-Q-TOF-MS/MS, Zebrafish, Molecular docking

## Abstract

**Background:**

*Bletilla striata* is the main medicine of many skin whitening classic formulas in traditional Chinese medicine (TCM) and is widely used in cosmetic industry recently. However, its active ingredients are still unclear and its fibrous roots are not used effectively. The aim of the present study is to discover and identify its potential anti-melanogenic active constituents by zebrafish model and molecular docking.

**Methods:**

The antioxidant activities were evaluated by 2,2-diphenyl-1-picrylhydrazyl (DPPH) radical scavenging activity, 2,2′-azino-bis-(3-ethylbenthiazoline-6-sulphonic acid) (ABTS) radical scavenging activity and ferric reducing antioxidant power (FRAP) assay*.* The anti-melanogenic activity was assessed by tyrosinase inhibitory activity in vitro and melanin inhibitory in zebrafish. The chemical profiles were performed by ultra-high-performance liquid chromatography combined with quadrupole time-of-flight tandem mass spectrometry (UPLC-Q-TOF-MS/MS). Meanwhile, the potential anti-melanogenic active constituents were temporary identified by molecular docking.

**Results:**

The 95% ethanol extract of *B. striata* fibrous roots (EFB) possessed the strongest DPPH, ABTS, FRAP and tyrosinase inhibitory activities, with IC_50_ 5.94 mg/L, 11.69 mg/L, 6.92 mmol FeSO_4_/g, and 58.92 mg/L, respectively. In addition, EFB and 95% ethanol extract of *B. striata* tuber (ETB) significantly reduced the melanin synthesis of zebrafish embryos in a dose-dependent manner. 39 chemical compositions, including 24 stilbenoids were tentatively identified from EFB and ETB. Molecular docking indicated that there were 83 (including 60 stilbenoids) and 85 (including 70 stilbenoids) compounds exhibited stronger binding affinities toward tyrosinase and adenylate cyclase.

**Conclusion:**

The present findings supported the rationale for the use of EFB and ETB as natural skin-whitening agents in pharmaceutical and cosmetic industries.

**Supplementary Information:**

The online version contains supplementary material available at 10.1186/s12906-021-03492-y.

## Background

*Bletilla striata* (Thunb.) Reichb. f. is a herbaceous perennial plant widely distributed in Asia, such as China, Korea, and Japan [1]. The dried tuber of *B. striata*, also known as Baiji, firstly recorded in Shennong’s Classic of Materia Medica, has been widely used as a traditional Chinese medicine (TCM) for thousands of years in China. Chinese pharmacopeias states that it possesses the capability of astringency upon hemostasis and analgesis, therefore, it was widely used for the treatment of hematemesis, hemoptysis, traumatic bleeding, ulcers, swelling and chapped skin [2, 3]. Pharmacological studies demonstrated that *B. striata* possessed a wide spectrum of biological activities, such as wound healing [4, 5], anti-ulcer [6, 7], hemostatic [8], anti-inflammation [9], antioxidant [10], antibacterial [11], anti-influenza viral [12], and antiaging [13]. At the same time, *B. striata* contains various classes of chemical compositions, including polysaccharides [14], bibenzyls, phenanthrenes, anthraquinones, flavonoids, and 2-isobutylmalates [3, 15], etc.

*B. striata* is the main medicine of many skin whitening classic formulas in TCM [16] and it is widely used in cosmetic industry recently [17]. However, its active ingredients are still unclear and even some research results were contrary. For instance, the research results given by Chen et al. indicated that 95% ethanol extract of *B. striata* possessed higher tyrosinase inhibitory activity than those of its water extract with inhibition rate of 68.36% in vitro [18], while the result of Huang et al. showed that the inhibitory activity of *B. striata* water extract on tyrosinase was stronger than that of 95% ethanol extract with inhibition rate of 62% in vitro [19]. The research results by Lu et al. [20] and Linghu et al. [21] demonstrated that both water and 95% ethanol extract of *B. striata*, especially its chloroform fractionation, could inhibit B16 cell growth and induce its apoptosis in a concentration-dependent manner.

As the in vitro tests of tyrosinase inhibition and melanoma cell lines, didn’t involve the complex physiological in vivo conditions, and the absorption, metabolism, distribution and excretion of the test sample, many of the samples showed significant inhibitory activity against tyrosinase and melanoma cell lines in vitro, nevertheless, exhibited weakly effective or even ineffective in vivo [22, 23]. The glabridin showed significant tyrosinase inhibitory activity with IC_50_ 0.43 μmol/L, which was 176 times stronger than that of kojic acid. Nevertheless, it was no inhibitory effects on the pigmentation of zebrafish in vivo [24].

Meanwhile, *B. striata* fibrous roots (FB) were the by-products generated during the processing of *B. striata* tuber (TB). Modern researches indicated that the FB contains similar compounds with TB and with higher content of phenolic [25]. In addition, the antibacterial [26], antioxidant and anti-tyrosinase activities of FB extract were stronger than those of TB extract [25]. However, the resources of FB were not effectively used and were abandoned in the farmland, which led to the waste of FB resources and environmental pollution [27].

Zebrafish (*Danio rerio*), a small tropical freshwater fish, is an emerging animal model for pharmacology and toxicology research in vivo, with many advantages including low cost, short life cycle, high transparency, easy to maintain, high fertility, and required fewer test samples [28, 29]. In addition, zebrafish has melanin pigments on the surface, allowing simple observation of the pigmentation process, and was widely used in the anti-melanogenic study [28, 30].

In the present study, we compared the antioxidant and tyrosinase inhibitory activities of crude polysaccharide and 95% ethanol extract of TB and FB in vitro, and anti-melanogenic activities in zebrafish model. Meanwhile, the potential anti-melanogenic active constituents were temporary identified by molecular docking. We discovered that 95% ethanol extract of TB (ETB) and FB (EFB) possesses the significantly antioxidant and anti-tyrosinase activities, and can reduce melanin synthesis of zebrafish embryos in a dose-dependent manner. Thus, ETB and EFB can be used as natural skin-whitening agents in pharmaceutical and cosmetic industries.

## Methods

### Chemicals and reagents

Tyrosinase, 3-(3,4-dihydroxyphenyl)-L-alanine (L-DOPA) and arbutin were purchased from Aladdin reagent company (Shanghai, China). 2,2-diphenyl-1-picrylhydrazyl (DPPH), 2,2′-azino-bis-(3-ethylbenthiazoline-6-sulphonic acid) (ABTS), 6-hydroxy-2,5,7,8-tetramethylchroman-2-carboxylic acid (Trolox), and 2,4,6-tris (2-pyridyl)-s-triazine (TPTZ) were purchased from Sigma-Aldrich (St. Louis, MO, USA). Acetonitrile and methanol (HPLC grade) were purchased from Tedia (Fairfield, OH, USA). Deionized water was prepared by the Milli-Q water system (18.2 MΩ, Millipore, USA).

### Plant collection and extraction procedure

*B. striata* was collected from Quzhou YiNianTang Agriculture and Forestry Technology Co., Ltd. (Quzhou, Zhejiang, China). Voucher specimens were deposited at Herbarium of Zhejiang Pharmaceutical College (Accession no. 190615). The whole plant was divided into tubers and fibrous roots. Subsequently, they were cut into small pieces and dried by vacuum freeze-drying, respectively.

The dried and powdered samples (TB and FB) were reflux extracted with 95% ethanol for three times (1.5 h for each time). The extract was filtered using a Whatman filter paper (No.1). The filtrate was concentrated to dryness in a rotary evaporator (Hei-VAP, Heidolph, Germany) at 50 °C and was subseqently dried by a vacuum freeze dryer. The ethanol extraction of TB (ETB) and FB (EFB) yields were 5.21 and 6.53%, respectively. The dregs were used to extract the crude polysaccharide by dispersed in 80 °C water for 4 h and precipitation with ethanol [31]. The polysaccharide yields of TB (PTB) and FB (PFB) were 14.75 and 6.45%, respectively.

### Chemical analysis

The chemical analysis was performed on an ultra-high-performance liquid chromatography combined with quadrupole time-of-flight tandem mass spectrometry (UPLC-Q-TOF-MS/MS). The chromatographic separation was performed on a Waters Acquity UPLC™ system (Waters Corp., Milford, MA, US) with a Waters BEH Shield RP C18 column (100 × 2.1 mm, 1.7 μm) at 30 °C. The mobile phase was consisted of 0.1% formic acid in water (A) and acetonitrile (B), with a linear gradient: 0–3 min, 5–16% B; 3–8 min, 16–30% B; 8–10 min, 30–35% B; 10–15 min, 35–55% B; 15–18 min, 55–80% B; 18–19 min, 80–5% B; 19–20 min, 5% B. The flow rate was 0.3 mL/min, and the injection volume was 2 μL.

The TOF-MS/MS experiments were performed by using an AB SCEIX Triple TOF 5600 mass spectrometry (AB SCIEX, Foster City, CA, USA) equipped with an electrospray ionization (ESI). The MS detection was conducted in negative ionization method. The parameters were set as follows: source and desolvation temperature were 100 °C and 450 °C respectively; desolvation gas flow rate was 900 L/h; capillary voltage was 2 kV; cone voltage was 40 V; collision energy was 22 eV; and the full scan spectra was from 100 to 2000 Da.

### Antioxidant assays

#### DPPH radical scavenging activity

The DPPH radical scavenging activity was determined in accordance with Ali et al. with slightly modifications [32]. Briefly, 50 μL test sample solution was mixed with 150 μL DPPH ethanol solution (0.2 mM) in 96-well plates and was kept in the dark for 30 min. The absorbance of the mixture at 517 nm (A_1_) was assessed by microplate spectrophotometer (Thermo Fisher Scientific, America). The blank solution without test sample mixed with DPPH ethanol solution was set as the positive group (A_0_). The blank solution without DPPH mixed with test sample solution was set as the blank group (A_2_). All experiments were executed in triplicate. The DPPH radical scavenging rate was calculated using the following formula:

DPPH radical scavenging rate (%) = [A_0_-(A_1_-A_2_)]/A_0_ × 100%.

#### ABTS radical scavenging activity

The ABTS radical scavenging capacity was measured using the method described by Ali et al. [32]. Briefly, 7 mM ABST aqueous solution was mixed with 2.45 mM potassium persulfate at a ratio of 1:1 (*v*/*v*), and incubating at room temperature in the dark for 16 h to obtain the ABST^+^ stock solution. The stock solution was diluted with phosphate buffered saline (PBS) to adjust the absorbance of (0.72 ± 0.2) at 734 nm. 20 μL test samples at different concentrations were mixed thoroughly with 180 μL ABTS^+^ solution. The reactive mixtures were kept in the dark for 5 min and subsequently measured the absorbance (B_1_) at 734 nm. The absorbance of the mixtures without test sample and ABST^+^ were set as B_0_ and B_2_, respectively. All experiments were executed in triplicate. The ABTS radical scavenging rate was calculated according to the following formula:

ABTS radical scavenging rate (%) = [B_0_-(B_1_-B_2_)]/B_0_ × 100%.

#### Ferric reducing antioxidant power assay (FRAP)

The ferric iron reducing activity was determined according the procedures by Kosakowska et al. [33]. 300 mM acetate buffer, 10 mM TPTZ (2,4,6-tris (2-pyridyl)-s-triazine) and 20 mM FeCl_3_ were mixed at a (*v*/*v*/*v*) ratio of 10:1:1 to prepared the working reagent. 100 μL of each test sample solution was mixed with 100 μL TPTZ working reagent, incubated at 37 °C for 20 min and the absorbance was determined at 593 nm. Meanwhile, a series of FeSO_4_ standard solutions in the concentration ranges of 0–1000 μg/mL were used to prepare the calibration curve. The ferric reducing capacity was calculated in the formation of an intense Fe^2+^-TPTZ blue complex. The results were expressed as Fe^2+^ antioxidant capacity (mmol FeSO_4_/g of extract).

### Tyrosinase inhibitory activity

Tyrosinase inhibitory activities were determined by spectrophotometric method using *L*-DOPA as substrate [34]. Briefly, 50 μL sample solutions at different concentrations were mixed with 50 μL tyrosinase (200 units/mL, dissolved in pH 6.8 phosphate buffer) in 96-well plates and incubated for 15 min at 25 °C. The reaction was then initiated with the addition of *L*-DOPA (50 μL). After incubating of 30 min at 25 °C, the absorbance at 490 nm (A_a_) was determined using a multiskan sky microplate spectrophotometer (Thermo Fisher Scientific, America). Similarly, the absorbance of the sample wells without tyrosinase (A_b_) and the control wells with enzyme but without sample (A_b_) were detected at the same time. The tyrosinase inhibitory activity was calculated by the equation as below:

Tyrosinase inhibitory rate (%) = [1− (A_a_ − A_b_)/A_c_] × 100%.

The IC_50_ was calculated using the GraphPad Prism® equation as below:

$$\mathrm{Y}=\min +\frac{\mathit{\max}-\mathit{\min}}{1+{10}^{\left(x-{logIC}_{50}\right)\times Hill\ slope}}$$

*X* represented the inhibitor concentration; Y represented the inhibition data (%) along with their minimal (min) and maximal (max) values; Hill slope is the slope factor.

### Melanin inhibitory in zebrafish

Wild-type AB line zebrafish (provided by Hunter Biotechnology, Inc., Hangzhou, Zhejiang Province) were housed in fish water (0.2% instant ocean salt in deionized water, pH 6.9–7.2, conductivity 480–510 mS/cm, and hardness 53.7–71.6 mg/L CaCO_3_) in a 14/10 h light/dark photoperiod at a constant temperature 28 ± 0.5 °C, and fed live brine shrimp twice daily. The zebrafish embryos were obtained from natural spawning and collected within 30 min. The zebrafish assay was accredited by the Association for Assessment and Accreditation of Laboratory Animal Care (AAALAC). The present study was approved by the IACUC (Institutional Animal Care and Use Committee) at Hunter Biotechnology, Inc. and the IACUC approval number was 001458.

Embryos at the 6 h post fertilization (hpf) stage were treated each extract at six concentrations (10, 30, 62.5, 125, 250 and 500 mg/L) for 72 h to evaluating the maximum non-lethal concentration (MNLC). As a result, the MNLCs of all the extract were more than 62.50 mg/L. 10 embryos were placed in 6-well and were exposed to tested samples at concentrations of 10 and 30 mg/L from 6 hpf to 54 hpf (48 h exposure). DMSO (0.05%, *v*/*v*) and arbutin (10 and 30 mg/L) were used as the normal and positive control, respectively.

Synchronized embryos were collected and observed under a stereomicroscope (SZX7, Olympus, Japan) equipped with a digital camera (VertA1, China). Melanin accumulation, directly correlated to the zebrafish pigmentation areas, was calculated using the GNU Image Manipulation Program (GIMP version 2.10.2) and expressed as percentage with respect to the negative control (melanin accumulation 100%) [28, 30].

### Molecular docking study

158 compounds isolated from *B. striata* in the literature [2], including 19 glycosides, 28 bibenzyls, 19 phenanthrenes, 18 biphenanthrenes, 23 dihydrophenanthrenes, 5 anthocyanins, 11 steroids, 8 triterpenoids, 12 phenolic acids, 5 quinones, and 10 other compounds, were used as the ligands. The 3D structures were drawn by ChemBio3D and were optimized using MM2 and Autodock Tools. The 3D crystal structure of tyrosinase (PDB ID:5M8N) and adenylate cyclase (PDB ID:5IV3) were retrieved from RCSB Protein Data Bank (www.rcsb.org/pdb/home/home.do). All water molecules and hetero atoms were removed from the crystal structures by using Chimera and MGL Tools. Finally, Autodock Vina was used to dock the receptor protein with the small molecule ligands. Parameters of the receptor protein docking site were set as − 36.700, 7.034, − 19.104, and − 17.467, − 22.807, 2.786, respectively, according to the original ligand mimosine and LRE1 [35]. In order to achieve higher computational accuracy, 20 exhaustiveness parameters for each receptor were generated, and the conformation with the highest affinity was selected as the final docking conformation and visualized in Pymol 2.3. Meanwhile, the original ligands mimosine and LRE1 were taken as the positive control [36].

### In silico ADMET prediction

The top 3 hits from *B. striata* possessed better binding affinity with tyrosinase and adenylate cyclase, were subjected to ADMET analysis. QikProp in Schrodinger suite was used to calculate their physical properties and drug-related characteristics, including molecular weight (MW), predicted octanol/water partition coefficient (QPlogPo/w), predicted aqueous solubility (QPlogS), predicted apparent Caco-2 cell permeability for gut-blood barrier (QPPCaco), predicted brain/blood partition coefficient (QPlogBB), predicted skin permeability (QPlogKp), predicted IC_50_ for blockage of HERG K^+^ channels (QPlog HERG) and predicted human oral absorption. The properties were assessed based on Lipinski’s rule of five and literature [37, 38].

### Molecular dynamics simulation

In order to examine the stability and dynamic fluctuations of the ligand-protein complex under a simulated biological environment, and further validate the docking results, the compounds blestrin D (**75**) was chosen as the ligand for the molecular dynamics (MD) simulation study, based on the molecular docking result. The complex of blestrin D with tyrosinase and adenylate cyclase were performed MD simulations and run for 100 ns, using TIP3P water mode. The root mean square deviation (RMSD) values of protein backbone atoms relative to the initial structure were calculated to examine the protein stability over the course of simulation period [39].

## Results

### Chemical composition

**Fig. 1:**
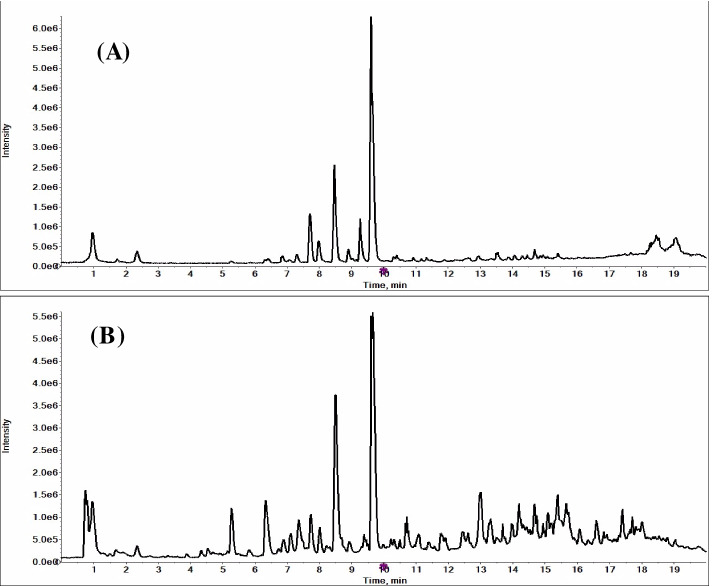
Base peak chromatogram of 95% ethanol extracts from *B. striata* tubers (**A**) and fibrous roots (**B**)

The typical base peak chromatogram chromatograms of ETB and EFB were shown in Fig. 1. The PeakView™ software was used to identify the constituents. The molecular formula was accurately assigned within mass error of 10 ppm. The exact molecular weight and the fragment ions were used to identify the components according the literatures and the free chemical structure database, such as ChemSpider and Massbank. As a result, 39 chemical compositions, including 24 stilbenoids (bibenzyls, phenanthrenes and their derivatives), 6 glycosides, 4 phenolic acids, 3 quinones, 1 steroid, and 1 other compound were tentatively identified from EFB and ETB. At the same time, their relative semi-quantification was performed by measuring peak areas of each compound in MS mode using the extracted ion chromatograms [40]. The detailed information of the identification and their relative contents (heat map highlights, the darker the color, the higher the concentration) were summarized in Table 1.

**Table 1:** The compounds identified from the 95% ethanol extracts from *B. striata* tubers and fibrous roots by UPLC-Q-TOF-MS/MS, and their relative peak areas

NO.	T_R_ (min)	Formula	Found Mass	[M + H]^−^ (Da)	Error (ppm)	Identification	Relative peak areas (× 10^6^)	
							ETB	EFB
1	2.35	C_13_H_18_O_7_	285.0977	161.0450, 123.0444, 105.0352	−0.8	gastrodin	2.17	1.43
2	4.53	C_7_H_6_O_3_	137.0248	137.0244, 108.0224, 92.0275	3.1	*p*-hydroxybenzoic acid	–	0.77
3	6.70	C_9_H_8_O_3_	163.0406	145.8896, 119.0505, 93.0361	3.2	3-hydroxycinnamic acid	–	0.43
4	7.01	C_34_H_32_O_8_	567.2028	457.1678, 393.1388, 285.0978, 161.0454, 153.0573, 129.0579	0.6	bleochranol D	–	2.80
5	7.71	C_40_H_56_O_22_	887.3179	707.2520, 619.2221, 439.1590	−1.3	dactylorhin A	3.93	3.06
6	8.13	C_21_H_22_O_8_	401.1228	284.0313, 255.0290, 238.0630, 227.0337, 195.0465	−3.6	2,7-dihydroxy-4-methoxyphenanthrene-2-O-glucoside	–	–
7	8.21	C_27_H_32_O_13_	563.1747	540.6504, 429.8704, 394.1452, 320.7833, 310.8648	−4.1	2,7-dihydroxy-4-methoxyphenanthrene-2,7-O-diglucoside	–	1.18
8	9.62	C_34_H_46_O_17_	725.2635	457.1673, 285.0954, 171.0654, 153.0551, 123.0451	−3.8	militarine	30.48	38.39
9	9.76	C_20_H_22_O_6_	357.1332	313.0688, 225.0513, 181.0614, 121.0287, 77.0403	−3.3	pinoresinol	–	–
10	10.31	C_21_H_26_O_8_	405.1537	243.1015, 227.0705, 201.0893, 136.0531, 122.0400	−4.5	3′-hydroxy-5-methoxybibenzyl-3-O-*β*-D-glucopyranoside	0.72	1.56
11	11.08	C_16_H_12_O_5_	283.0601	237.0654, 211.0864, 75.0467	−3.8	1,8-dihydroxy-3-methoxy-6-methylanthracene-9,10-dione	–	1.53
12	12.15	C_15_H_13_O_4_	256.0734	239.0345, 211.0401, 201.8356, 167.0496, 143.0501	−3.0	7-hydroxy-2-methoxyphenanthrene-3,4-dione	–	–
13	12.42	C_15_H_12_O_3_	239.0714	224.0466, 196.0523, 167.0491	0.3	4-methoxyphenanthrene-2,7-diol	–	1.92
14	12.99	C_16_H_14_O_4_	269.0820	254.0563, 211.0388, 183.0448	0.2	2,7-dihydroxy-3,4-dimethoxyphenanthrene	–	6.01
15	13.54	C_51_H_64_O_24_	1059.3674	791.2752, 661.2301, 569.1989, 439.1569, 153.0557	−3.8	gymnoside X	1.07	0.89
16	13.64	C_28_H_26_O_5_	441.1691	347.1255, 253.0852, 241.0868, 211.0751, 93.0360	−3.7	shanciguol	–	1.51
17	13.80	C_16_H_18_O_4_	273.1126	243.1036, 227.0700, 185.0636, 136.0516, 122.0372, 106.0415	−2.2	3,3′-dihydroxy-5,4′-dimethoxybibenzyl	–	–
18	13.88	C_15_H_16_O_3_	243.1026	227.0707, 183.0810, 136.0537, 93.0357	−0.4	3,3′-dihydroxy-5-methoxybibenzyl	–	1.80
19	14.07	C_22_H_20_O_4_	347.1278	332.1024, 237.0561, 225.0549, 209.0622, 93.0352	−3.2	4,7-dihydroxy-1-(*p*-hydroxybenzyl)-2-methoxy-9,10-dihydrophenanthrene	0.32	–
20	14.26	C_29_H_24_O_5_	451.1507	437.1341, 392.1347, 329.0777, 224.0484	−9.8	1,8-*bis*(*p*-hydroxybenzyl)-4-methoxyphenanthrene-2,7-diol	–	8.73
21	14.31	C_22_H_18_O_4_	345.1124	330.0875, 302.0909, 237.0545	−2.4	1-(*p*-hydroxybenzyl)-4-methoxyphenanthrene-2,7-diol	0.25	–
22	14.50	C_25_H_24_O_6_	419.1480	405.1118, 377.1172, 225.0548	−4.8	bleochranol B	0.21	1.89
23	14.67	C_23_H_20_O_5_	375.1225	360.0979, 317.0802, 224.0484	−3.6	bleformin B	–	5.08
24	14.71	C_30_H_26_O_6_	481.1631	465.1301, 434.1102, 419.1264, 225.0548	−5.3	blestrin A	0.79	5.08
25	14.89	C_22_H_22_O_4_	349.1434	243.1017, 227.0704, 183.0810, 93.0356	−3.1	3,3′-dihydroxy-4-(*p*-hydroxybenzyl)-5-methoxybibenzyl	–	–
26	14.94	C_30_H_24_O_6_	479.1478	464.1236, 432.0980, 421.1041, 379.0948, 238.0631, 224.0478, 196.0533	−4.6	blestrin D	0.46	1.91
27	15.53	C_32_H_26_O_8_	537.1529	522.1312, 507.1027, 492.0773, 464.0849, 421.0668, 377.0799, 209.8293, 130.9680	−4.9	4,8,4′,8′-tetramethoxy-[1,1′-biphenanthrene]-2,7,2′,7′-tetrol	0.01	9.15
28	15.74	C_37_H_32_O_7_	587.2047	571.1716, 543.1831, 527.1544, 476.1243, 449.1367, 434.1257, 270.2400, 224.0534, 152.9984	−4.9	bleformin D	–	9.63
29	16.08	C_15_H_14_O_3_	241.0869	226.0615, 183.0449, 169.0667	−0.7	2,4-dimethoxyphenanthrene-7-ol	0.13	1.48
30	16.49	C_27_H_26_O_7_	461.1584	425.1723, 381.1463, 242.0897, 93.0338	−4.7	pleionesin C	0.04	0.36
31	16.61	C_16_H_14_O_3_	253.0862	238.0621, 223.0388, 195.0441, 167.0502	−3.1	2-hydroxy-4,7-dimethoxyphenanthrene	0.01	1.42
32	16.89	C_18_H_18_O_4_	297.1126	253.0491, 239.0346, 225.0942, 211.0392, 166.0456	−2.1	2,3,4,7-tetramethoxyphenanthrene	0.01	2.28
33	16.94	C_29_H_26_O_5_	453.1671	438.1428, 345.1106, 251.0712, 195.0470, 93.0362	−8.2	2,7-dihydroxy-1,6-bis(*p*-hydroxybenzyl)-4-methoxy-9,10-dihydrophenanthrene	0.10	0.64
34	17.46	C_23_H_24_O_4_	363.1585	333.1163, 255.0940, 227.0706, 199.0746, 157.0631, 93.0378	−4.7	bulbocol	0.15	3.85
35	17.67	C_22_H_22_O_3_	333.1486	163.1126, 107.0521, 75.0477	−3.2	5-hydroxy-2-(*p*-hydroxybenzyl)-3-methoxybibenzyl	0.60	3.34
36	18.27	C_27_H_35_O_9_	502.2211	369.2416, 337.2141, 193.1229, 163.1124, 147.0816, 133.1056	0.5	(20*S*,22*R*)-1*β*,2*β*,3*β*,4*β*,5*β*,7*α*-hexahydroxyspirost-25 (27)-en-6-one	9.14	1.06
37	18.30	C_18_H_30_O_3_	293.2117	275.1976, 249.1821, 235.1714, 221.1501, 171.1030, 121.1041, 59.0153	−1.7	striatolide	–	2.49
38	18.78	C_16_H_32_O_2_	255.2327	238.027, 224.0464, 210.0314, 195.0444, 182.0355, 167.0502	−1.0	palmitic acid	–	0.95
39	18.87	C_31_H_23_O_8_	522.1329	508.1470, 493.1272, 450.1078, 253.0495, 225.0527, 210.0426	1.7	3′,7′,7-trihydroxy-2,2′,4′-trimethoxy-[1,8′-biphenanthrene]-3,4-dione	7.20	0.76
**Total**							**57.79**	**123.37**

- means the relative peak areas is less than 0.01 × 10^6^

### Antioxidant capacity

**Fig. 2:**
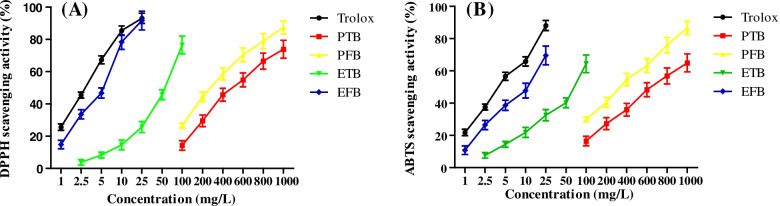
The DPPH (**A**) and ABTS (**B**) radical scavenging activities of PTB, PFB, ETB, EFB, and Trolox

It is well known that Ultraviolet A (UVA)-irradiation can induce reactive oxygen species (ROS) production and mediate excessive melanogenesis in skin cells. Natural polyphenol was the inhibitors of ROS generation and could be responsible for the anti-melanogenic activity of plant extracts [41, 42]. Thus, in the present study, the antioxidant capacity was assessed through DPPH, ABTS radical scavenging activity and FRAP assay. The results (Table 2 and Fig. 2) showed that the EFB (IC_50_ = 5.94 mg/L) possesses the strongest DPPH radical scavenging activity in vitro compared to the PTB (IC_50_ = 548.24 mg/L), PFB (IC_50_ = 285.81 mg/L), and ETB (IC_50_ = 65.25 mg/L). A similar trend was observed in ABTS and FRAP assay. The 95% ethanolic extracts of TB and PB had stronger antioxidation activity in vitro than their crude polysaccharides. In addition, EFB exhibit stronger antioxidation activity than ETB, which was consistent with the previous research results [25].

**Table 2:** The results of antioxidant activity analyses in vitro (DPPH, ABTS and FRAP)

Sample	IC_50_ values (mg/L)		FRAP mmol FeSO_4_/g
	DPPH scavenging	ABTS scavenging	
PTB	548.24 ± 8.93^a^	626.49 ± 9.75^a^	0.11 ± 0.01^a^
PFB	285.81 ± 5.31^b^	348.62 ± 6.30^b^	0.42 ± 0.03^b^
ETB	65.25 ± 1.85^c^	78.40 ± 2.51^c^	2.45 ± 0.05^c^
EFB	5.94 ± 0.46^d^	11.69 ± 0.64^d^	6.92 ± 0.10^d^
Trolox	3.52 ± 0.27^e^	3.68 ± 0.31^e^	–

Within a row, different letters (a, b, c, d and e) indicate significant differences with *p* < 0.05

### Tyrosinase inhibitory activity

**Fig. 3:**
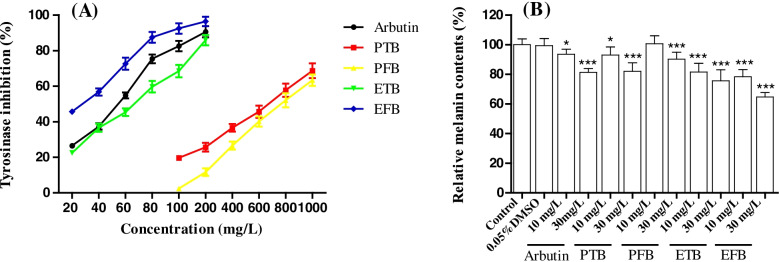
Impact of melanogenic inhibitors on relative tyrosinase activity (**A**) in vitro and melanin synthesis (**B**) in zebrafish embryos

Tyrosinase was the key rate-limiting enzyme in melanin biosynthesis pathway and was widely used to identify the potential of natural products with anti-melanogenesis effect [43]. The results (Fig. 3) showed that ETB and EPB had stronger tyrosinase inhibitory activity than PTB and PPB in vitro, which was consistent with previous research results [25]. EFB exhibited stronger tyrosinase inhibition activity in a dose dependent manner (between 20 and 200 mg/L) with IC_50_ = 58.92 mg/L than ETB (IC_50_ = 75.44 mg/L) and the positive compound arbutin (IC_50_ = 66.02 mg/L).

### Melanin inhibitory in zebrafish

**Fig. 4:**
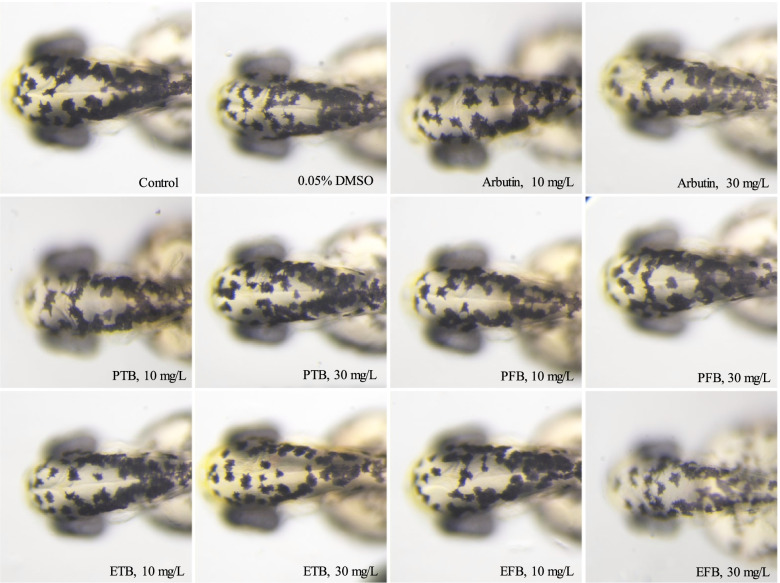
The impacts of PTB, PFB, ETB and EFB on the melanin synthesis of zebrafish embryos (× 2 × 10 × 5.6)

Zebrafish are recognized as a highly advantageous vertebrate model system to evaluate anti-melanogenesis activity, as it possesses similar organ systems and gene sequences to human beings [28]. Therefore, zebrafish was used to evaluate the anti-melanogenesis activity. As shown in Fig. 4, a large number of melanin (black spots) deposited in the zebrafish embryo in the control and 0.05% DMSO group. The microscopy images showed there was no significant difference in total melanin content between two groups (*p* > 0.05), indicating that the vehicle 0.05% DMSO didn’t affect the melanin production in zebrafish embryos. However, after exposure to the tested samples and arbutin for 48 h, zebrafish embryos exhibited varying degrees of melanin deposition (Figs. 3B, 4), excepting PFB 10 mg/L group. It is noticeable that the relative melanin contents in ETB 10 mg/L (78.38%) and 30 mg/L (64.72%) groups were significant lower than that in PTB 10 mg/L (93.06%) and 30 mg/L (82.02%) groups (*p* < 0.01). It demonstrated that the anti-melanogenesis activity of ETB was superior to that of PTB, which was consistent with the tyrosinase inhibitory activity. Whereafter, it is noteworthy that the anti-melanogenesis activities of ETB (81.65, 75.61%) and EFB (78.38, 64.72%) were stronger than that of arbutin (93.57, 81.48%) at the same dose of 10 mg/L and 30 mg/L (*p* < 0.01 or 0.05).

### Molecular docking

Tyrosinase is the rate-limiting enzyme in melanin biosynthesis: hydroxylation of tyrosine to 3,4-dihydroxyphenylalanine (DOPA), and oxidation of DOPA to dopaquinone. Therefore, tyrosinase has been considered as a critical target for the development of melanogenesis inhibitors [44]. Additionally, adenylate cyclase is key enzyme of cAMP-induced melanogenesis. The binding of melanotropin alpha polypeptide (α-MSH) to MC1R receptors induced the activation of adenylate cyclase, increase in the cAMP level, up-regulated expression of tyrosinase gene, and subsequently increase the melanin synthesis [45]. Therefore, we performed a molecular docking study of the 158 compounds isolated from *B. striata* previously [2], with tyrosinase and adenylate cyclase. The binding energies of the studied ligands with tyrosinase and adenylate cyclase were summaried in Table S1. There were 83 (including 60 stilbenoids) and 85 (including 70 stilbenoids) compounds exhibit stronger binding affinities toward tyrosinase and adenylate cyclase, comparing to those of the original ligands mimosine (− 6.4 kcal/mol) and LRE1 (− 8.5 kcal/mol). 1,8-bis(*p*-hydroxybenzyl)-4-methoxyphenanthrene-2,7-diol (**56**), blestrin D (**75**), and 2,7-dihydroxy-1,6-bis(*p*-hydroxybenzyl)-4-methoxy-9,10-dihydrophenanthrene (**93**) were the top three ligands toward tyrosinase, with binding energy − 10.2, 10.0, and 9.7 kcal/mol, respectivey. While blestrin D (**75**), blestrin B (**73**), and 3,3′-dihydroxy-5-methoxy-2,5′,6-tris(*p*-hydroxybenzyl) bibenzyl (**42**) were the top three ligands toward adenylate cyclase, with binding energy − 12.1, − 11.9, and − 11.6 kcal/mol, respectivey. Their theoretical binding affinities and ligand-amino acid interactions were summaried in Table 3.

**Table 3:** Summary of binding affinities and ligand-amino acid interactions

Protease	Ligand	Binding Energy (kcal/mol)	H-Bond	lipophilic
Tyrosinase	1,8-bis(p-hydroxybenzyl)-4-methoxyphenanthrene-2,7-diol	−10.2	–	Lys233, Leu229, Arg114, Glu451, Arg230, Pro115, Gly107, Pro445, Met452, Tyr226, Ser106, Asn459
	blestrin D	−10.0	Arg114, Glu451	Pro446, Pro445, Ser106, Gly107, Cys113, Lys233, Arg230, Pro115, Tyr226, Leu229, Met452
	2,7-dihydroxy-1,6-bis(p-hydroxybenzyl)-4-methoxy-9,10dihydrophenanthrene	−9.7	Glu232, Gln236, Lys223, Cys113, Glu451, Ser106,	Leu229, Ile128, Pro115, Tyr226, Val447, Gly107, Thr112
Adenylate cyclase	blestrin D	−12.1	Val167	Leu166, Lys95, Phe45, Ala97, Asn412, Arg416, Ala415, Val172, Phe336, Phe338, Arg176, Leu102, Met337
	blestrin B	−11.9	Met337, Val167	Met419, Phe338, Phe45, Lys95, Leu166, Phe165, Phe336, Leu102, Ala97, Phe296, Ala415, Arg416
	3,3′,5-trimethoxybibenzyl	−11.6	Asp47, Asn180	Ala100, Leu345, Ala97, Phe336, Phe296, Met419, Ala415, Met418, Lys95, Leu166, Val335, Phe165, Leu102, Phe45, Phe338, Arg176, Gln179

**Fig. 5:**
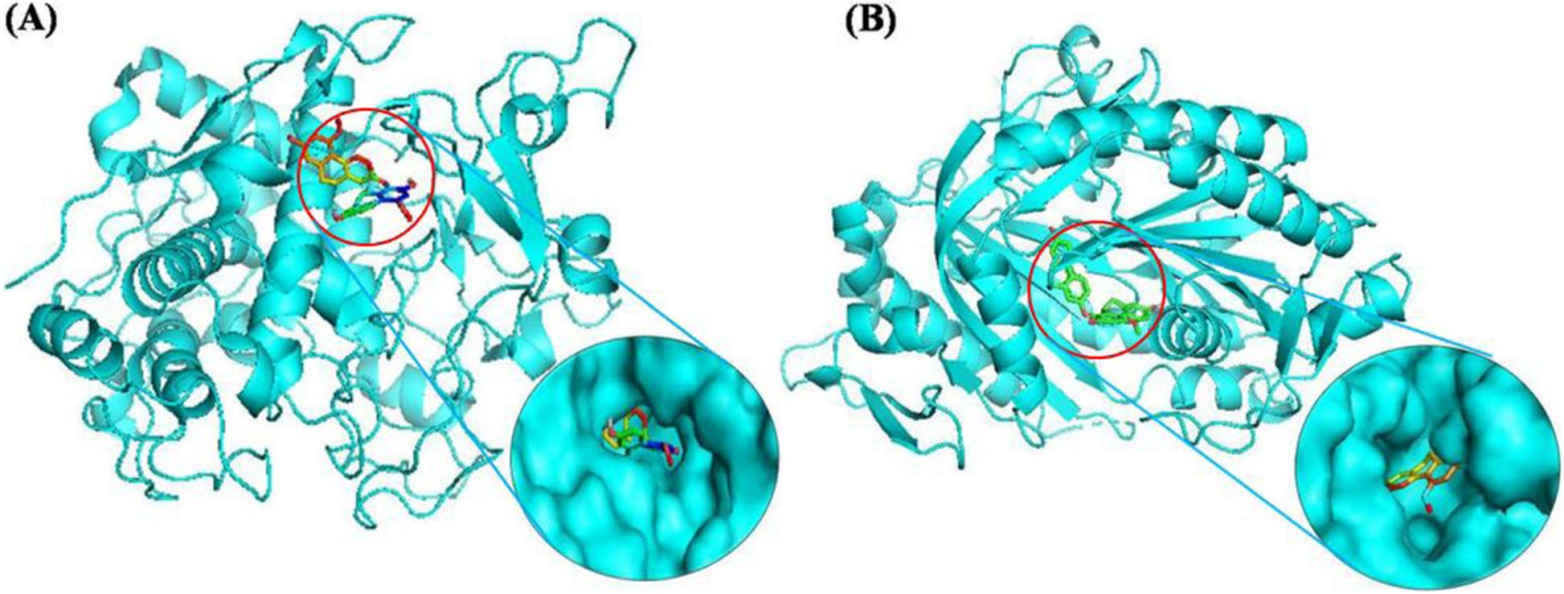
Best calculated poses for blestrin D inside binding pocket of homology model of tyrosinase (**A**) and adenylate cyclase (**B**)

It was worth noting that blestrin D (75), a biphenanthrene compound, entered into the binding pocket of tyrosinase and adenylate cyclase (Fig. 5), and exhibited significant binding affinities toward both tyrosinase and adenylate cyclase. The Van der Walls interactions contributes to overall energy interaction value, meanwhile hydroxyl group produces hydrogen bonds with amino acid residues Glu 451, Arg114 of tyrosinase, and Val 167 of adenylate cyclase, respectivey (Table 3 and Fig. S1). Compound **93** also exhibited significant binding affinities toward tyrosinase through formation of 6 hydrogen bonds with amino acid residues Glu232, Glu451, Ser106, Cys113, Lys223, and Gln236.

### In silico ADMET prediction

The predicted values of several important parameters along with their acceptable range were summarized in Table 4. Apart from the QPlogS of 3,3′,5-trimethoxybibenzyl, blestrin B and blestrin D, and QPlogBB of 3,3′,5-trimethoxybibenzyl, all the other calculated ADMET properties of the top three hits were within the expected ranges, meanwhile, the QPlog HERG of all the top three hits were less than − 5, indicating they were drugable potential for human use. The QPlog Kp of all the top three hits for both tyrosinase and adenylate cyclase, excepting 3,3′,5-trimethoxybibenzyl, were in the favorable range, indicating these compounds can easily penetrate into skin.

**Table 4:** Qikprop calculated ADMET properties of the top three hits

Compounds	MW	QPlog Po/w	QPlogS	QPP Caco	QP logBB	QPlog Kp	QPlog HERG	Human oral absorption (%)	Rule of five
3,3′,5-trimethoxybibenzyl	272.34	4.31	− 6.02	9906.04	− 0.15	− 0.15	−7.77	100.00	2
1,8-bis(p-hydroxybenzyl)-4-methoxyphenanthrene-2,7-diol	452.51	4.70	−6.25	149.72	−1.98	−2.81	−6.66	93.41	0
blestrin B	482.53	5.55	−7.26	661.17	−1.17	−2.10	−6.29	96.94	1
blestrin D	482.53	5.50	−7.47	452.82	−1.39	−2.43	−6.40	93.71	1
2,7-dihydroxy-1,6-bis(p-hydroxybenzyl)-4-methoxy-9,10dihydrophenanthrene	348.40	3.70	−4.91	375.43	−1.23	−2.73	−5.50	94.67	0

Recommended values: MW, 130.0–725.0; QPlogPo/w, −2 - 6.5; QPlogS, −6.5 - 0.5; QPPCaco < 25 is poor and > 500 is great; QPlogBB, −3 - 1.2; QPlogKp,–8.0 - –1.0; QPlog HERG, <− 5; human oral absorption (%) > 80% is high and < 25% is poor; rule of five, max 4

### Molecular dynamic simulation

**Fig. 6:**
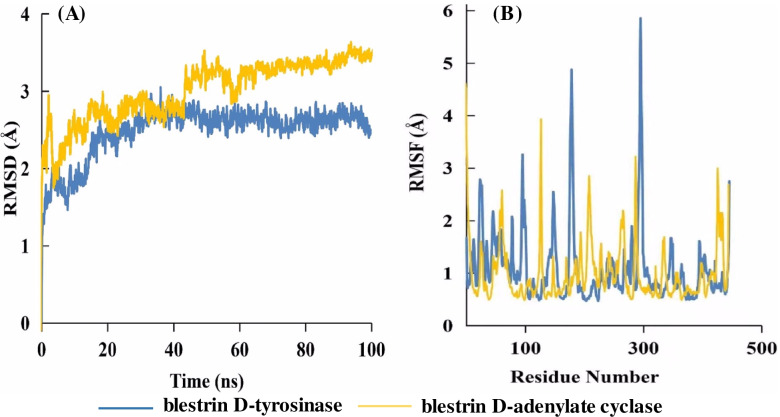
Dynamics of blestrin D bound to tyrosinase and adenylate cyclase. (A) The backbone RMSD of the protein along the simulation time. (B) RMSF for protein-inhibitor during MD simulation

**Fig. 7:**
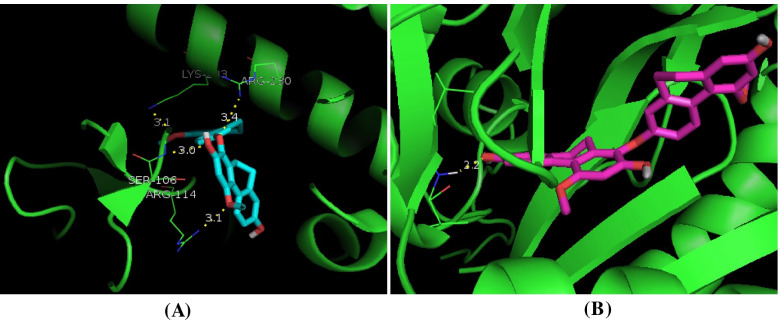
Molecular interactions of blestrin D with tyrosinase (A) and adenylate cyclase (B). Dashed lines represent the H-bonds

The RMSD plot showed that the RMSD values of the protein-ligand complex did not fluctuate significantly throughout the entire simulation period. The RMSD values (Fig. 6A) of blestrin D with tyrosinase and adenylate cyclase complex were determined ranging from 1.5 to 2.8 Å, and from 2.0 to 3.6 Å, respectively. The RMSF value reflecting the flexibility of each residue during simulations. The residues with higher RMSF values revealed they were more flexible (Fig. 6B). The RMSF values of the amino acid residues in loop regions were found to highly fluctuate. While, the active site residues kept a small RMSF value (less than 1.0 Å), such as Arg114, Val167, Tyr226, Leu229, and Arg230 in tyrosinase, and Val167, Leu102, Phe45, Lys95 and Leu166 in adenylate cyclase, indicating the binding pockets were kept stable. The molecular interactions of blestrin D with tyrosinase and adenylate cyclase were shown in Fig. 7.

The binding free energies were calculated by molecular mechanics-generalized born surface area (MM-GBSA) method [39]. The predicted binding free energy of blestrin D with tyrosinase and adenylate cyclase were − 96.94 kcal/mol and − 139.69 kcal/mol, respectively, implying the binding interactions were spontaneous (Table 5). Furthermore, the contributions favoring ligand binding were nonpolar solvation interaction, van der Waals and electrostatic interaction.

**Table 5:** Binding free energy (kcal/mol) of inhibitor-protein complexes along with the individual energy components (kcal/mol) contributions

Contribution	Blestrin D-tyrosinase	Blestrin D-adenylate cyclase
△G_VDW_	−54.03	−72.34
△G_ele_	−11.48	−28.68
△G_GB_	30.44	37.98
△G_GA_	−61.71	−78.47
△G_bind_	−96.94	−139.69

△G_VDW_: The free energy of binding from the van der Waals energy, △G_ele_: The free energy of binding from the electrostatic energy, △G_GB_: The free energy of binding from the polar solvation energies, △G_GA_: The free energy of binding from the non-polar solvation, △G_bind_: Free energy of binding

## Discussion

It was worth noting that the types and contents of the chemical components in EFB were more than that in ETB. The peak areas of all the identified compounds in EFB were approximately two-fold of that in ETB. Militarine was the most abundant compounds in both EFB and ETB, which possessed significant antioxidant, anti-inflammatory and neuroprotection activities, and was chosen as the chemical markers for the quality control of *B. striata* in Chinese Pharmacopoeia 2020 edition [46]. The relative peak area of militarine in EFB (38.39 × 10^6^) was slightly higher than that in ETB (30.48 × 10^6^). Gastrodin is a well-known compound in many Chinese herbal medicines, such as *Gastrodia elata*, which exhibits significant tyrosinase inhibitory and radical scavenging effects [30]. The relative peak area of gastrodin in EFB (1.43 × 10^6^) was slightly lower than that in ETB (2.17 × 10^6^).

The 95% ethanolic extracts of TB and PB had stronger antioxidation activity in vitro than their crude polysaccharides. In addition, EFB exhibit stronger antioxidation activity than ETB, which was consistent with the previous research results [25]. This phenomenon may be attributed to that the EFB contains higher level of antioxidant compounds, such as militarine and stilbenoids (natural plant polyphenols). For example, the relative peak area of 4,8,4′,8′-tetramethoxy-[1,1′-biphenanthrene]-2,7,2′,7′-tetrol, a biphenanthrene with four phenolic hydroxyl groups, was 9.15 × 10^6^ in FB, while it was 0.01 × 10^6^ in TB.

In the present study, PPB and PTB showed slight tyrosinase inhibition activity in a dose dependent manner. However, it demonstrated that no activity was detected in the aqueous extract of PB (contains PPB) [25]. This phenomenon may be due to the different sample preparation. The aqueous extract of PB was used to the evaluate tyrosinase inhibitory activity in Jiang’s experiment [25], while in the present study, aqueous extract was precipitation with ethanol to obtain the crude polysaccharide.

Recently, the zebrafish model widely used in evaluation of anti-melanogenesis effect in vivo [47, 48]. Arbutin, a hydroquinone glucoside compound existing in various plants, which has been commercially used in the cosmetic industry, was set as positive control. The anti-melanogenesis activities of ETB and EFB were stronger than that of arbutin, indicating that ETB and EFB can be applied as skin-lightening agents in cosmetic industry.

The relative peak area of blestrin D in EFB (1.91 × 10^6^), is approximately four times of that in ETB (0.46 × 10^6^). The relative peak area of compound **56** (8.73 × 10^6^) and **93** (0.64 × 10^6^) in EFB is also significant higher than that in ETB (less than 0.01 × 10^6^ and 0.10 × 10^6^). This may be one of the reasons why EFB exhibits stronger anti-melanogenic effects than ETB. It is interesting to note that top three ligands toward tyrosinase and adenylate cyclase all belong to stilbenoids (bibenzyls, phenanthrenes and its derivative). Stilbenoids are the natural plant polyphenols, which has attracted great interest in the last years because of their remarkable bioactivities such as anti-inflammatory, antimicrobial and antioxidant activity [49]. Resveratrol is the most common stilbenoid. The previous research indicated that resveratrol and its derivatives can significantly inhibit the catalytic activity, gene expression, and posttranslational modifications of tyrosinase. Thus, they showed potentially useful as skin lightening and antiaging agents in cosmetics [41, 50].

## Conclusions

In this study, the antioxidant and anti-melanogenic activity of the crude polysaccharide from the *B. striata* tuber (PTB) and fibrous roots (FPB)*,* and 95% ethanol extract of *B. striata* tuber (ETB) and fibrous roots (EFB) were systematically investigated. The results showed that EFB possessed the strongest DPPH, ABTS radical scavenging activity and ferric iron reducing activity. In addition, ETB and EFB can significantly reduce tyrosinase activity in vitro and melanin synthesis of zebrafish embryos in a dose-dependent manner. Molecular docking indicated that there were a large number of compounds, mostly belonged to stilbenoids, exhibiting stronger binding affinities toward tyrosinase and adenylate cyclase, compared to the binding affinity of the original ligand. The present findings supported the rationale for the use of ETB and EFB as natural skin-whitening agents in pharmaceutical and cosmetic industries.

## Supplementary Information

**Additional file 1.**

**Additional file 2.**

## Data Availability

The datasets used and/or analysed during the current study are available from the corresponding author on reasonable request.

## Abbreviations

TCM: Traditional Chinese medicine
TB: *Bletilla striata* tuber
FB: *Bletilla striata* fibrous roots
ETB: 95% ethanol extract of *Bletilla striata* tuber
EFB: 95% ethanol extract of *Bletilla striata* fibrous roots
PTB: Crude polysaccharide of *Bletilla striata* tuber
PFB: Crude polysaccharide of *Bletilla striata* fibrous roots
DPPH: 2,2-Diphenyl-1-picrylhydrazyl
TPTZ: 2,4,6-Tris (2-pyridyl)-s-triazine
FRAP: Ferric reducing antioxidant power assay
ABTS: 2,2′-Azino-bis-(3-ethylbenthiazoline-6-sulphonic acid
Trolox: 6-Hydroxy-2,5,7,8-tetramethylchroman-2-carboxylic acid
L-DOPA: 3-(3,4-Dihydroxyphenyl)-L-alanine
UPLC-Q-TOF-MS/MS: Ultra-high-performance liquid chromatography combined with quadrupole time-of-flight tandem mass spectrometry
MNLC: Maximum non-lethal concentration
ROS: Reactive oxygen species
MW: Molecular weight
QPlogPo/w: Predicted octanol/water partition coefficient
QPlogS: Predicted aqueous solubility
QPPCaco: Predicted apparent Caco-2 cell permeability for gut-blood barrier
QPlogBB: Predicted brain/blood partition coefficient
QPlogKp: Predicted skin permeability
QPlog HERG: Predicted IC_50_ for blockage of HERG K^+^ channels
MD: Molecular dynamics
RMSD: Random mean square deviation
MM-GBSA: Molecular mechanics-generalized born surface area
RMSF: Root mean square fluctuation
△G_VDW_: The free energy of binding from the van der Waals energy
△G_ele_: The free energy of binding from the electrostatic energy
△G_GB_: The free energy of binding from the polar solvation energies
△G_GA_: The free energy of binding from the non-polar solvation
△G_bind_: Free energy of binding
