# Supplementary material for: Discovery and identification of potential anti-melanogenic active constituents of Bletilla striata by zebrafish model and molecular docking
Source: BMC Complement Med Ther. 2022 Jan 7;22:9. doi: 10.1186/s12906-021-03492-y (PMC8742349; doi:10.1186/s12906-021-03492-y)
Supplement: Supplementary file 1 — Additional file 1. [file 12906_2021_3492_MOESM1_ESM.pdf]

Table S1. The docking scores of 158 compounds with tyrosinase and adenylate cyclase.

NO.	Compound	Formula	Binding Energy (kcal/mol)	
			Tyrosinase	Adenylate cyclase
1	dactylorhin A	$C_{40}H_{56}O_{22}$	4.6	-6.3
2	gymnoside I	$C_{21}H_{30}O_{11}$	-5.3	-8.9
3	gymnoside II	$C_{21}H_{30}O_{11}$	-7.8	-8.1
4	gymnoside V	$C_{49}H_{62}O_{23}$	-8.5	-8.8
5	gymnoside IX	$C_{51}H_{64}O_{24}$	23.7	-4.8
6	gymnoside X	$C_{51}H_{64}O_{24}$	-	-
7	militarine	$C_{34}H_{46}O_{17}$	-	-
8	bletilnoside A	$C_{38}H_{62}O_{12}$	-4.2	-8.5
9	bletilnoside B	$C_{38}H_{60}O_{12}$	3.8	-3.8
10	3-O-β-D-glucopyranosyl-3-epiruscogenin	$C_{41}H_{60}O_{12}$	3.5	-4.3
11	3-O-β-D-glucopyranosyl-3-epineoruscogeni n	$C_{41}H_{58}O_{12}$	26.4	-7
12	dancosterol	$C_{35}H_{60}O_{6}$	24.4	-5.1
13	2,7-dihydroxy-4-methoxyphenanthrene-2-O -glucoside	$C_{21}H_{22}O_{8}$	-5.2	-7.1
14	2,7-dihydroxy-4-methoxyphenanthrene-2,7-O-diglucoside	$C_{27}H_{32}O_{13}$	-9.0	-10.0
15	3,7-dihydroxy-2,4-dimethoxyphenanthrene-3-O-glucoside	$C_{22}H_{24}O_{9}$	-0.7	-8.5
16	gastrodin	$C_{13}H_{18}O_{7}$	-9.4	-8.7
17	2,7-dihydroxy-l-(4’-hydroxybenzyl)-9,10-di hydrophenan-threne-4’-O-glucoside	$C_{28}H_{30}O_{9}$	-7.1	-6.7
18	3’-hydroxy-5-methoxybibenzyl-3-O-β-D-gl ucopyranoside	$C_{21}H_{26}O_{8}$	-7.0	-11.3
19	blestritin A	$C_{37}H_{36}O_{6}$	-8.8	-9.6
20	blestritin B	$C_{30}H_{30}O_{6}$	-3.6	-10.1
21	blestritin C	$C_{36}H_{34}O_{6}$	-4.9	-10.0
22	bulbocodin	$C_{36}H_{34}O_{6}$	-4.6	-10.5
23	bulbocodin C	$C_{29}H_{28}O_{5}$	-9.1	-11.1
24	bulbocodin D	$C_{29}H_{28}O_{5}$	-9.3	-9.7
25	bulbocol	$C_{23}H_{24}O_{4}$	-9.2	-10.1
26	gymconopin D	$C_{23}H_{24}O_{4}$	-9	-9.7
27	shancigusin B	$C_{28}H_{26}O_{5}$	-8.9	-9.6
28	shanciguol	$C_{28}H_{26}O_{5}$	-6.2	-10.1
29	arundinan	$C_{22}H_{22}O_{3}$	-8.2	-10
30	arundin	$C_{29}H_{28}O_{4}$	-8.7	-9.5
31	batatasin III	$C_{15}H_{16}O_{3}$	-7.2	-10.2

32	gigantol	$C_{16}H_{18}O_4$	-8.2	-8.6
33	3,4'-dihydroxy-5,3',5'-trimethoxybibenzyl	$C_{17}H_{20}O_5$	-8.4	-8.2
34	3,3'-dihydroxy-5,4'-dimethoxybibenzyl	$C_{16}H_{18}O_4$	-8.3	-8.3
35	3'-O-methylbatatasin III	$C_{15}H_{18}O_3$	-8.5	-8.2
36	3,3'-dihydroxy-4-(p-hydroxybenzyl)-5-methoxybibenzyl	$C_{22}H_{22}O_4$	-8.2	-8.6
37	3,3'-dihydroxy-2-(p-hydroxybenzyl)-5-methoxybibenzyl	$C_{22}H_{22}O_4$	-8.5	-9.7
38	3',5-dihydroxy-2-(p-hydroxybenzyl)-3-methoxybibenzyl	$C_{22}H_{22}O_4$	-8.9	-9.5
39	2',6'-bis(p-hydroxybenzyl)-5-methoxybibenzyl-3,3'-diol	$C_{33}H_{36}O_5$	-9.0	-9.8
40	2,6-bis(p-hydroxybenzyl)-5,3'-dimethoxybibenzyl-3-ol	$C_{30}H_{30}O_5$	-7.5	-10.4
41	3,3'-dihydroxy-5-methoxy-2,5',6-tris(p-hydroxybenzyl) bibenzyl	$C_{46}H_{44}O_{11}$	-6.9	-10.2
42	3,3',5-trimethoxybibenzyl	$C_{17}H_{20}O_3$	-4.3	-11.6
43	3,5-dimethoxybibenzyl	$C_{16}H_{18}O_2$	-8.0	-8.5
44	5-hydroxy-4-(p-hydroxybenzyl)-3',3-dimethoxybibenzyl	$C_{23}H_{24}O_4$	-7.6	-8.4
45	3,3'-dihydroxy-5-methoxybibenzyl	$C_{15}H_{16}O_3$	-8.5	-9.7
46	5-hydroxy-2-(p-hydroxybenzyl)-3-methoxy bibenzyl	$C_{22}H_{22}O_3$	-8.2	-8.6
47	4-methoxyphenanthrene-2,7-diol	$C_{15}H_{12}O_3$	-8.8	-9.6
48	3,4-dimethoxyphenanthrene-2,7-diol	$C_{16}H_{14}O_4$	-8.6	-9.0
49	2,4-dimethoxyphenanthrene-3,7-diol	$C_{16}H_{14}O_4$	-8.4	-8.3
50	3,5-dimethoxyphenanthrene-2,7-diol	$C_{16}H_{14}O_4$	-8.1	-9.2
51	1,5-dimethoxyphenanthrene-2,7-diol	$C_{16}H_{14}O_4$	-7.5	-8.3
52	2,4-dimethoxyphenanthrene-7-ol	$C_{15}H_{14}O_3$	-8.7	-8.5
53	2,4,7-trimethoxyphenanthrene	$C_{17}H_{16}O_3$	-8.1	-9.0
54	2,3,4,7-tetramethoxyphenanthrene	$C_{18}H_{18}O_4$	-7.5	-8.9
55	2,3,4,7-tetramethoxyphenanthrene	$C_{18}H_{18}O_4$	-7.2	-8.1
56	1,8-bis(p-hydroxybenzyl)-4-methoxyphenanthrene-2,7-diol	$C_{29}H_{24}O_5$	-10.2	-10.7
57	1-(p-hydroxybenzyl)-4,8-dimethoxyphenanthrene-2,7-diol	$C_{23}H_{20}O_5$	-9.2	-9.1
58	1-(p-hydroxybenzyl)-4-methoxyphenanthre	$C_{22}H_{18}O_4$	-9.5	-10.5

	ne-2,7-diol			
59	2-hydroxy-4,7-dimethoxyphenanthrene	$C_{16}H_{14}O_3$	-7.5	-8.7
60	3,7-dihydroxy-2,4,8-trimethoxyphenanthrene	$C_{17}H_{16}O_5$	-8.4	-7.8
61	2,7-dihydroxy-3,4-dimethoxyphenanthrene	$C_{16}H_{14}O_4$	-8.4	-8.3
62	1-(p-hydroxybenzyl)-4,7-dimethoxyphenanthrene-2-ol	$C_{23}H_{20}O_4$	-9.0	-10.5
63	1-(p-hydroxybenzyl)-4,7-dimethoxyphenanthrene-2,8-diol	$C_{23}H_{20}O_5$	-8.4	-9.4
64	1-(p-hydroxybenzyl)-4,7-dimethoxyphenanthrene-2,6-diol	$C_{23}H_{20}O_5$	-9.1	-10.2
65	bleformin B	$C_{23}H_{20}O_5$	-9.3	-8.7
66	blespirol	$C_{25}H_{18}O_5$	-6.1	-10.3
67	1,8-dihydroxy-3-methoxy-6-methylanthracene-9,10-dione	$C_{16}H_{12}O_5$	-9.3	-9.0
68	2-methylanthraquinone	$C_{15}H_{10}O_2$	-9z.0	-10.4
69	4,7-dimethoxyphenanthrene-1,2-dione	$C_{16}H_{13}O_4$	-7.7	-9.0
70	7-hydroxy-2-methoxyphenanthrene-3,4-dione	$C_{15}H_{13}O_4$	-9.1	-9.9
71	3’,7’,7-trihydroxy-2,2’,4’-trimethoxy-[1,8’-biphenanthrene]-3,4-dione	$C_{31}H_{23}O_8$	2.2	-8.5
72	blestrin A	$C_{30}H_{26}O_6$	-6.9	-11.1
73	blestrin B	$C_{30}H_{26}O_6$	-9.6	-11.9
74	blestrin C	$C_{30}H_{24}O_6$	-6.9	-11.2
75	blestrin D	$C_{30}H_{24}O_6$	-10	-12.1
76	blestriarene A	$C_{30}H_{26}O_6$	-5.0	-7.1
77	blestriarene B	$C_{30}H_{24}O_6$	-3.9	-7.9
78	blestriarene C	$C_{30}H_{22}O_6$	-5.3	-9.5
79	blestrianol A	$C_{30}H_{26}O_6$	-5.4	-10.2
80	blestrianol B	$C_{37}H_{32}O_7$	0.2	-10.6
81	blestrianol C	$C_{37}H_{30}O_7$	4.6	-8.0
82	4,7,3’5’-tetramethoxy-9’,10’-dihydro-[1,2’-biphenanthrene]-2,7’- diol	$C_{32}H_{27}O_6$	6.5	-9.8
83	4,7,7’-trimethoxy-9’,10’-dihydro-[1,3’-biphenanthrene]-2,2’,5’- triol	$C_{31}H_{25}O_6$	0.8	-8.6
84	4,7,4’-trimethoxy-9’,10’-dihydro-[1,1’-biphenanthrene]-2,2’,7’- triol	$C_{31}H_{25}O_6$	-3.4	-8.2

85	4,7,3’,5’-tetramethoxy-9’,10’-dihydro-[1,1’-biphenanthrene]- 2,2’,7’-triol	$C_{32}H_{27}O_7$	-2.7	-8.5
86	4,8,4’,8’-tetramethoxy-[1,1’-biphenanthrene]-2,7,2’,7’-tetrol	$C_{32}H_{26}O_8$	1.1	-7.4
87	bleformin D	$C_{37}H_{32}O_7$	3.7	-8.5
88	4,4’-dimethoxy-9,10-dihydro-[6,1’-biphenanthrene]-2,7,2’,7’-tetraol	$C_{30}H_{24}O_6$	-5.5	-10.9
89	gymconopin C	$C_{30}H_{26}O_6$	-5.3	-10.9
90	4,7-dihydroxy-2-methoxy-9,10-dihydrophenanthrene	$C_{15}H_{14}O_3$	-8.8	-9.2
91	2,7-dihydroxy-3-(p-hydroxybenzyl)-4-methoxy-9,10dihydrophenanthrene	$C_{22}H_{20}O_4$	-9.3	-10.2
92	4,7-dihydroxy-1-(p-hydroxybenzyl)-2-methoxy-9,10dihydrophenanthrene	$C_{22}H_{20}O_4$	-9.7	-9.6
93	2,7-dihydroxy-1,6-bis(p-hydroxybenzyl)-4-methoxy-9,10dihydrophenanthrene	$C_{29}H_{26}O_5$	-9.7	-9.6
94	2,7-dihydroxy-l,3-bis(p-hydroxybenzyl)-4-methoxy-9,10dihydrophenanthrene	$C_{29}H_{26}O_5$	-6.8	-10.2
95	2,7-dihydroxy-l-(p-hydroxybenzyl)-4-methoxy-9,10dihydrophenanthrene	$C_{22}H_{20}O_4$	-8.8	-10.8
96	2,4,7-trimethoxy-9,10-dihydrophenanthrene	C17H18O3	-7.3	-8.9
97	2,7-dihydroxy-4-methoxy-9,10-dihydrophenanthrene	$C_{15}H_{14}O_3$	-8.6	-8.8
98	4,5-dihydroxy-2-methoxy-9,10-dihydrophenanthrene	$C_{15}H_{14}O_3$	-8.2	-9.1
99	2,8-dihydroxy-4,7-dimethoxy-9,10-dihydrophenanthrene	$C_{15}H_{14}O_3$	-8.7	-8.3
100	2,8-dihydroxy-1-(p-hydroxybenzyl)-4,7-dimethoxy-9,10dihydrophenanthrene	$C_{23}H_{22}O_5$	-8.3	-9.5

101	pleionesin C	$C_{27}H_{26}O_7$	-7.6	-9.3
102	(2,3-trans)-2-(4-hydroxy-3-methoxyphenyl) -3-hydroxymethyl-10methoxy-2,3,4,5-tetrahydro-phenanthro[2,1-b]furan-7-ol	$C_{25}H_{24}O_6$	-8.5	-9.7
103	bleochranol A	$C_{40}H_{38}O_8$	4.5	-8.6
104	bleochranol B	$C_{25}H_{24}O_6$	-9.3	-9.9
105	bleochranol C	$C_{33}H_{32}O_8$	-2.3	-9.6
106	bleochranol D	$C_{34}H_{32}O_8$	-3.9	-9.6
107	(2,3-trans)-3-[2-hydroxy-6-(3-hydro-xyphenethyl)-4-methoxybenzyl]-2-(4-hydroxy-3-methoxyphenyl)-10-methoxy-2,3,4,5-tetrahydrophenanthro[2,1-b]furan-7-ol	$C_{17}H_{18}O_4$	19.4	-9.0
108	shanciol	$C_{25}H_{24}O_6$	-5.8	-10.6
109	bletlos A	$C_{28}H_{28}O_8$	-3.1	-7.6
110	bletlos B	$C_{27}H_{26}O_7$	-3.9	-9.3
111	bletlos C	$C_{27}H_{26}O$	-6.1	-9.5
112	blestriaren A	$C_{30}H_{26}O_6$	-4.7	-7.7
113	Bletilla anthocyanin 1	$C_{75}H_{81}O_{40}$	106.3	16.6
114	Bletilla anthocyanin 2	$C_{72}H_{79}O_{37}$	99.9	29.7
115	Bletilla anthocyanin 3	$C_{75}H_{81}O_{43}$	112.7	46.7
116	Bletilla anthocyanin 4	$C_{72}H_{79}O_{40}$	106.3	36.6
117	3-O-(β-glucopyranoside)-7-O-[6-O-(4-O-(6-O-(4-O-(β-glucopyranosyl)-trans-caffeoyl)-β-glucopyranosyl)-trans-caffeoyl)β-glucopyranoside]	$C_{57}H_{63}O_{32}$	77.0	6.5
118	β-sitosterol	$C_{29}H_{50}O$	-5.4	-9.6
119	β-sitosterol palmitate	$C_{45}H_{80}O_2$	0.8	-6.8
120	stigamasterol	$C_{29}H_{48}O$	-4.6	-10.5
121	stigamasterol palmitat	$C_{45}H_{78}O_2$	4.4	-6.5
122	3-epiruscogenin	$C_{27}H_{42}O_4$	-0.4	-9.2
123	3-epineoruscogenin	$C_{27}H_{40}O_4$	-0.8	-7.3
124	(20S,22R)-1β,2β,3β,4β,5β,7α-hexahydroxyspirost-25(27)-en-6-one	$C_{27}H_{35}O_9$	4.3	-6.2
125	(1α,3α)-1-O-[(β-D-xylopyranosyl-(1→2)-α-L-rhamnopyranosyl)]-3-O-D-glucopyranosyl-5α-spirostan	$C_{44}H_{71}O_{17}$	43.2	-5.9
126	(1α,3α)-1-O-[(β-D-xylopyranosyl-(1→2)-α-L-rhamnopyranosyl)oxy]-3-O-D-glucopyranosyl-25(27)-ene-5α-spirostan	$C_{44}H_{69}O_{17}$	44.5	-2.4

127	(1α,3α)-1-O-[(β-D-xylopyranosyl-(1→2)-α-L-rhamnopyranosyl)oxy]-epiruscogenin	$C_{38}H_{59}O_{12}$	48.6	-2.1
128	(1α,3α)-1-O-[(β-D-xylopyranosyl-(1→2)-α-L-rhamnopyranosyl)oxy]-epineoruscogenin	$C_{38}H_{57}O_{12}$	35.5	-3.4
129	cyclomargenol	$C_{32}H_{54}O$	-0.7	-8.0
130	cyclomargenone	$C_{32}H_{53}O$	-0.6	-8.2
131	cycloneolitsol	$C_{32}H_{54}O$	-0.7	-9.0
132	cyclobalanone	$C_{32}H_{53}O$	0.8	-8.1
133	24-methylenecycloartanol palmitate	$C_{47}H_{81}O_2$	7.7	-6.7
134	cyclolaudenol	$C_{31}H_{51}O$	-1.4	-8.5
135	cyclolaudenone	$C_{31}H_{50}O$	0.5	-9.4
136	3β-hydroxyoleane-12-en-28-oic acid 3-O-α-L-rhamnopyranosyl-(1→2)-β-D-glucopyranoside	$C_{41}H_{55}O_{12}$	13.7	-1.7
137	p-hydroxybenzoic acid	$C_7H_6O_3$	-5.9	-6.0
138	protocatechuic acid	$C_7H_6O_4$	-6.3	-6.1
139	cinnamic acid	$C_9H_8O_2$	-6.5	-7.0
140	caffeic acid	$C_9H_8O_4$	-6.8	-7.0
141	2-hydroxysuccinic acid	$C_4H_5O_5$	-5.2	-4.4
142	palmitic acid	$C_{16}H_{32}O_2$	-5.7	-6.7
143	syringaresinol	$C_{22}H_{26}O_8$	-9.0	-8.5
144	pinoresinol	$C_{20}H_{22}O_6$	-9.2	-9.4
145	3’’-methoxynyasol	$C_{18}H_{17}O_3$	-8.5	-8.9
146	p-hydroxybenzaldehyde	$C_7H_6O_2$	-5.6	-5.4
147	ferulic acid	$C_{10}H_{10}O_4$	-6.7	-7.0
148	3-hydroxycinnamic acid	$C_9H_8O_3$	-6.7	-6.9
149	4-hydroxybenzylamine	C_7H_9NO	-5.5	-5.4
150	4,4’-dihydroxydiphenylmethane	$C_{13}H_{12}O_2$	-7.3	-7.7
151	4,4’-dihydroxybenzyl sulfide	$C_{14}H_{14}SO_2$	-7.2	-8
152	5-(hydroxymethyl)-2-furaldehyde	$C_6H_6O_3$	-5.8	-5.2
153	striatolide	$C_{18}H_{30}O_3$	-7.2	-8.4
154	schizandrin	$C_{24}H_{32}O_7$	-1.3	-7.0
155	brugnanin	$C_{56}H_{90}O_8$	13.0	-5.5
156	bletillanol A	$C_{18}H_{21}O_5$	-8.4	-8.3
157	bletillanol B	$C_{18}H_{20}O_5$	-8.2	-7.6
158	tupichinol A	$C_{17}H_{18}O_4$	-8.2	-8.0
	mimosine		-6.4	
	LER1			-8.5
