# Supplementary material for: Discovery and identification of potential anti-melanogenic active constituents of Bletilla striata by zebrafish model and molecular docking
Source: BMC Complement Med Ther. 2022 Jan 7;22:9. doi: 10.1186/s12906-021-03492-y (PMC8742349; doi:10.1186/s12906-021-03492-y)
Supplement: Supplementary file 2 — Additional file 2. [file 12906_2021_3492_MOESM2_ESM.pdf]

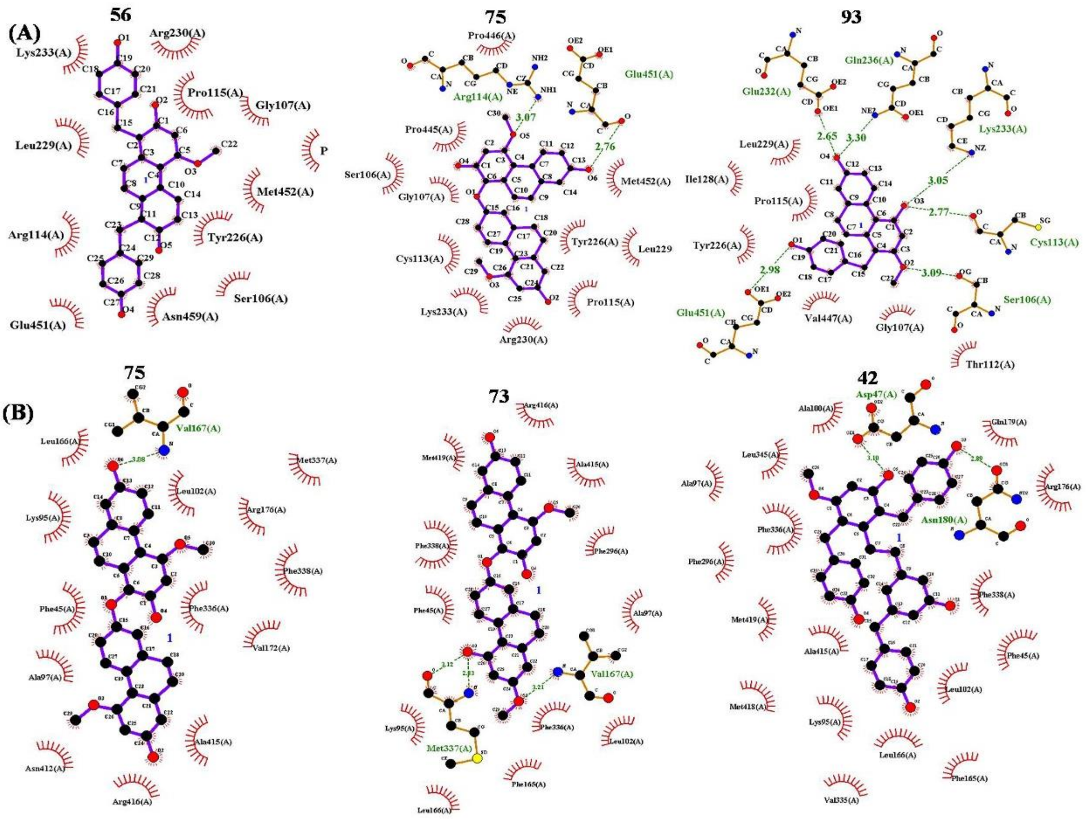

Fig. S1 Two-dimensional representation of interaction between studied ligands and amino acids inside binding pocket of homology model of (A) tyrosinase and (B) adenylate cyclase.
